# Molecular markers in bladder cancer

**DOI:** 10.1007/s00345-018-2503-4

**Published:** 2018-09-26

**Authors:** Francesco Soria, Laura-Maria Krabbe, Tilman Todenhöfer, Jakub Dobruch, Anirban P. Mitra, Brant A. Inman, Kilian M. Gust, Yair Lotan, Shahrokh F. Shariat

**Affiliations:** 1 Department of Urology and Comprehensive Cancer Center, Vienna General Hospital, Medical University of Vienna, Währinger Gürtel 18-20, 1090 Vienna, Austria; 2 Division of Urology, Department of Surgical Sciences, San Giovanni Battista Hospital, University of Studies of Torino, Turin, Italy; 3 Department of Urology, University of Texas Southwestern Medical Center, Dallas, TX USA; 4 Department of Urology, Eberhard-Karls-University, Teubingen, Germany; 5 Centre of Postgraduate Medical Education, Warsaw, Poland; 6 Institute of Urology, University of Southern California, Los Angeles, CA USA; 7 Duke Cancer Institute, Duke University, Durham, NC USA; 8 Karl Landsteiner Institute of Urology and Andrology, Vienna, Austria; 9 Department of Urology, Weill Cornell Medical College, New York, USA

**Keywords:** Bladder cancer, Urinary biomarkers, Tissue biomarkers, Blood biomarkers, Guidelines

## Abstract

**Purpose:**

Use of molecular markers in urine, tissue or blood offers potential opportunities to improve understanding of bladder cancer biology which may help identify disease earlier, risk stratify patients, improve prediction of outcomes or help target therapy.

**Methods:**

A review of the published literature was performed, without restriction of time.

**Results:**

Despite the fast-growing literature about the topic and the approval of several urinary biomarkers for use in clinical practice, they have not reached the level of evidence for widespread utilization. Biomarkers could be used in different clinical scenarios, mainly to overcome the limitations of current diagnostic, predictive, and prognostic tools. They have been evaluated to detect bladder cancer in asymptomatic populations or those with hematuria and in surveillance of disease as adjuncts to cystoscopy. There is also a potential role as prognosticators of disease recurrence, progression and survival both in patients with non-invasive cancers and in those with advanced disease. Finally, they promise to be helpful in predicting the response to local and/or systemic chemotherapy and/or immunotherapy.

**Conclusions:**

To date, due to the lack of high-quality prospective trials, the level of evidence provided by the current literature remains low and, therefore, the potential of biomarkers exceeds utilization in clinical practice.

## Introduction

Bladder cancer (BCa) is a heterogeneous disease with significant diagnostic, therapeutic and prognostic challenges [1]. The tools available to clinicians for diagnosis and staging require invasive procedures such as cystoscopy and biopsy in addition to imaging with computer tomography and magnetic resonance. These tools, however, often under-stage patients and lack sensitivity to detect all cancers (false negatives) and specifically micro-metastatic disease. These imprecisions in capturing the clinical and biologic potentials of a tumor result in over- and under-treatment with side effects of therapies [2].

Molecular markers detected in urine, tissue or blood offer promising opportunities to improve our understanding of biology of a specific cancer and its micro- and macroenvironment. This could help identify disease earlier, risk stratify patients, improve prognostication and prediction of outcomes and help target therapy. While some areas such as urine-based tumor markers have been more extensively studied, the current guidelines have yet to embrace markers in routine management of BCa [3]. In this consensus guideline, part of the SIU–ICUD update on BCa, we review the challenges of introducing markers into clinical care and discuss urine-, tissue- and blood-based markers for different stages of disease and different clinical scenarios. Evidence was selected through a non-systematic review of the literature.

## Challenges of marker introduction into clinical practice

Biomarker research can be categorized, similar to drug-development studies, into initial pre-clinical exploratory studies, clinical assay development and validation studies, small clinical retrospective studies, external validation in larger cohorts (retrospective or prospective, usually multi-institutional), prospective clinical trials and further post-approval studies as well as possible expansion to other clinical scenarios and disease stages [4, 5]. In an attempt to improve design, analysis and reporting of marker studies, a set of reporting recommendations has been developed and is generally accepted.

The main goal for marker development is to identify a validated test which can improve clinical decision-making in a cost-effective way. It is, therefore, not sufficient to merely show statistically significant independent association of the marker with the investigated outcome, but to show improved prognostic or predictive accuracy of a multivariable model over already available clinical features alone. Ideally, the integration of the marker should be improved with regard to discrimination, calibration and decision-analysis [5].

Finally, BCa is a heterogeneous disease. Therefore, it is unlikely that one single marker exists that can adequately characterize the potential and behavior of a cancer to allow reliable treatment conclusions. This has led many investigators to evaluate comprehensive pathways rather than single markers [6, 7]. Marker panels including drivers from key pathways in combination with clinical and pathological variables might be the most promising approach for accurate risk stratification and clinical decision-making for BCa.

## Biomarkers according to clinical stages

### Urinary biomarkers for screening and hematuria workup

In patients without history of BCa, there are several scenarios in which urinary biomarkers may play a role. One scenario that has frequently been discussed is the use of biomarkers for screening purposes. So far, the low prevalence of the disease in the general population has been a challenge for developing effective screening strategies [8]. Actually, the effectiveness of a screening program is significantly affected by the incidence and mortality of a specific disease. The number of trials applying biomarkers for BCa in a screening population is limited and, to date, there are no randomized controlled trials.

Identification of high-risk populations may help overcome these limitations [9]. However, data from recent trials indicate that even in patients with an increased risk of developing BCa such as workers exposed to occupational hazards with carcinogenic potential or heavy smokers, the incidence of BCa is too low for a broad screening to have a socioeconomic benefit. Lotan et al. [10], for example, assessed the value of NMP22 in a high-risk population including 1175 men and 327 women based on a history of at least 10 years of smoking or an occupational exposure of at least 15 years. Eighty-five (5.7%) subjects had a positive NMP-22. Three from the 69 patients undergoing further evaluation had abnormal findings (one pTa low-grade tumor, one pTa high-grade tumor and one atypia). During long-term follow-up, only nine additional patients were diagnosed with BCa [11]. Of note, no patient had muscle-invasive cancer (MIBC) and a positive NMP22 was not associated with worse overall survival (OS).

A second clinical scenario in which urinary biomarkers may add value is in the risk stratifying patients with asymptomatic microhematuria (AMH). Patients with asymptomatic gross hematuria have a significant risk of BCa (approximately 10%) making urologic workup necessary [12]. In these patients, urinary biomarkers could be used as an adjunct to cystoscopy and imaging but are currently unlikely to impact the diagnostic strategies. In patients with AMH, international guidelines differ significantly on the optimal workup. The prevalence of AMH in the adult population ranges as high as 18%, yet only 2% of the referred patients harbor BCa. This has led to a nonchalant general approach to AMH with limited workups and delayed referrals resulting in late diagnosis, especially in women [13]. In this setting, urinary biomarkers may help early identification of patients at risk triaging and fastening referral for a urologic workup [12, 14–16]. Cha et al. performed a retrospective analysis of 1182 patients with hematuria, including 68% with AMH and evaluated cytology, imaging, cystoscopy, and immunocytology in all patients. A nomogram predicting the risk of BCa was constructed showing a predictive accuracy of 90.8% [17]. Lotan et al. [15] validated a nomogram incorporating age, gender, ethnicity, smoking history, type of hematuria, cytology and NMP22 BladderChek in a cohort of 381 subjects with hematuria. In total, 23 patients (6.0%) had BCa. The predictive accuracy of the nomogram was 80.2%. Finally, in a cohort including 86 patients with AMH and 83 patients with gross hematuria, Beukert et al. [18] used methylation analysis of OSR1, SIM2, OTX1, MEIS1 and ONECUT2 for developing a model for prediction of BCa. The model also included clinicopathologic characteristics such as type of hematuria, age, gender and cytology results. The model yielded a sensitivity and specificity of 85% and 87% with an area under the curve (AUC) of 89%.

While these studies suggest a potential benefit to urinary biomarkers in patients with AMH, prospective controlled trials are lacking and urgently needed. Moreover, so far, there is no evidence that the use of urinary biomarkers in a screening/early detection setting has an effect on cancer-specific mortality [3]. Therefore, due to the low levels of evidence (LoE) provided, urinary biomarkers are currently not recommended for the screening of BCa or in prioritization of patients with AMH.

### Urinary biomarkers in surveillance setting

Urinary biomarkers in surveillance setting have been developed to overcome the limitations of cystoscopy and urine cytology. The former is invasive and may miss significant proportion of cancer recurrences, especially CIS, whereas the latter has low sensitivity in low-/intermediate-risk non-muscle-invasive bladder cancer (NMIBC) and suffers from considerable inter- and intra-observer variabilities especially in patients after BCG immunotherapy [19–21]. Biomarker implementation in surveillance may be categorized with two potential applications: (1) as an adjunct to cystoscopy and (2) its substitute. Furthermore, the role of a biomarker in clinical decision-making would be different in low-/intermediate-risk NMIBC and in high-risk NMIBC. In patients with low-grade disease it is possible that a marker could reduce the number of cystoscopies needed. For high-grade cancers, the marker would be an adjunct to cystoscopy and an abnormal result would increase awareness of patients and physicians, identify those at risk of progression, facilitate the interpretation of indeterminate results of cytology and assess response to BCG.

A urine marker would need a very high sensitivity and negative predictive value (NPV) to replace cystoscopy. One recent survey suggests that patient preference would have sensitivity as high as 90–95% for a marker to replace cystoscopy [22]. Overall, reported sensitivities of voided urine cytology, NMP22 (nuclear matrix protein), BTA (bladder tumor antigen) stat and BTA trak, Immunocyt, UBC test, Cyfra 21-1, FISH (fluorescence in situ hybridization) and Cxbladder Monitor in this setting differ widely between trials, ranging from 7% to 93% [23, 24]. Similarly, corresponding specificities range from 49% to 99% [25, 26]. Of these biomarkers, only Cxbladder Monitor has shown a high sensitivity (91–93%) while its rate of false-negative results does not exceed 1.5% [24]. Both metrics are believed to be prerequisites for the test to supplant cystoscopy. However, for low-grade tumors it may be less clinically relevant if a small tumor is missed by either cystoscopy or a marker.

Due to the unsatisfactory performances of most single biomarkers, panels of markers have been used to improve sensitivity or alternatively combining markers. One study found that combining two tests among cytology, immunocytology, FISH and NMP22 resulted in sensitivity and negative predictive value of no greater than 89.8% (Immunocyt + NMP22) and 92.1% (FISH + Immunocyt) [27]. If cytology is supplemented with any of the four tests, corresponding values are no greater than 86.7% (NMP22) and 91.3% (immunocytology). Adding FISH to conventional urine cytology is associated with 80.5% sensitivity (94.0% for high-risk tumors) and 90.1% negative predictive value (98.8% for high-risk tumors).

Based on these findings, the European (EAU), the American Urological Associations (AUA) as well as the Society of Urologic Oncology (SUO) do not recommend urinary biomarkers yet for the routine surveillance of patients with NMIBC [28, 29]. According to AUA/SUO Guideline “clinician may use biomarkers to assess response to intravesical BCG (UroVysion® FISH) and adjudicate equivocal cytology (UroVysion® FISH and ImmunoCyt®)” [29]. Actually, serial measurements of UroVysion FISH in patients subjected to BCG therapy revealed that abnormal test results at baseline (before BCG), at 6 weeks (before the 6^th^ BCG instillation) and before the 3 months’ cystoscopy (before the first maintenance course) are significantly associated with both cancer recurrence and progression. FISH at 3 months identified 50% of patients who experienced cancer progression within 2 years in half of those with positive test compared to only 3% of those who had normal result [30].

Another approach toward improving surveillance protocols of NMIBC and optimizing costs is to use reflex testing. In patients with negative/uncertain result of one test, the accuracy of follow-up is significantly increased by adding subsequent highly sensitive biomarkers. Immunotherapy is known to evoke inflammatory changes within the bladder often making reliable assessment of the lower urinary tract challenging. As such, accuracy of cytology used as an adjunct to cystoscopy to increase the detection of CIS or upper tract lesions is hampered by BCG. FISH and ImmunoCyt® were investigated in patients with atypical cytology. UroVysion FISH has 100% sensitivity and 100% negative predictive value in those with negative cystoscopy, yet equivocal cytology [31]. ImmunoCyt has 73% sensitivity in detecting recurrent bladder tumor in patients with atypical cytology with corresponding negative predictive value of 80% [32]. In summary, both tests are recognized by AUA/SUO as the potential reflex biomarkers to adjudicate atypical cytology to help avoid unnecessary workups.

In summary, in the surveillance of patients with NMIBC, there is insufficient evidence that urinary biomarkers can replace cystoscopy and prospective studies are necessary to demonstrate whether this is safe. Urinary biomarkers, however, could be used to help assess the response to intravesical immunotherapy and as reflex test in cases with equivocal urinary cytology (Expert Opinion).

### Tissue biomarkers for non-muscle-invasive bladder cancer

Tissue biomarkers can theoretically be used in NMIBC to predict oncological outcomes such as recurrence and progression as well as the response to intravesical BCG and, ideally, could be used to improve individualized treatment and surveillance based on individualized risk profiles. Moreover, they can be useful to identify the proportion of high-risk NMIBC patients who are likely to develop disease progression to invasive disease, thus requiring consideration for intensified therapy such as early radical cystectomy.

To date biomarkers associated with pathways important for tumor growth and spread have been evaluated intensively, such as cycle cell regulators, angiogenesis, apoptosis, signaling proteins and hormones.

p53, the product of TP53, the most common oncosuppressor gene mutated in all human cancers, has been associated with features of tumor aggressiveness and correlated with poor oncological outcomes [6, 33–37]. Moreover, it is associated with the most aggressive T1G3 cancers. Two meta-analyses summarizing the role of p53 in NMIBC showed that its overexpression can predict progression in T1HG patients but is not able to predict the response to BCG therapy [38, 39]. However, the heterogeneity of the included studies and limitations related to the immunohistochemistry hampered any clear conclusions [38].

The association between an altered expression of the tumor suppressor retinoblastoma (Rb) and oncological outcomes in BCa is relatively weak and the majority of trials failed to find an association with recurrence and progression [40]. More recently, it has been shown that Rb could be of predictive utility for BCa recurrence and progression only when combined with other biomarkers such as p53 and p27 [6, 34] in a panel of markers.

The antiapoptotic biomarker survivin was found to predict recurrence, progression and survival [41, 42]. In the largest series, Fristrup et al. [43] analyzed the expression of survivin in 283 NMIBC patients and reported a strong association with recurrence, progression and OS. Recently, a meta-analysis of 14 studies reported a statistically significant association with recurrence, cancer-specific survival (CSS), and OS [44]. However, prospective large series are lacking. Other biomarkers of apoptosis such as Livin, Bcl-2 and Bax have been investigated, but there are few studies [45, 46]. Finally, cell signaling pathway biomarkers such as ErbB and FGFR family members as well as angiogenesis (VEGF, MVD, HIF-1α) and tumor cell invasion biomarkers (E-cadherin and N-cadherin) have been shown to be related to outcomes of NMIBC [47, 48].

Based on the current literature, one can conclude that none of the evaluated tissue biomarkers alone could be used to predict oncological outcomes with sufficient accuracy to change decisions in routine clinical practice. Therefore, it has been postulated that a panel of biomarkers could improve the predictive accuracy over clinical information alone [49, 50]. However, even in this setting, results are conflicting and, therefore, to date, due to the low level of evidence and to the contrasting reported findings, the use of tissue biomarkers in BCa is not recommended since it does not change/improve clinical decision-making.

### Blood and tissue biomarkers for invasive bladder cancer

#### Tissue-based biomarkers

The development of MIBC involves alterations in multiple homeostatic pathways with profound deregulations within a complex molecular circuitry. Therefore, these alterations can serve as prognosticators of outcomes, predictors of response to therapy, and they may also act as therapeutic targets.

Several retrospective studies have reported that nuclear accumulation of p53 is prognostic in MIBC, especially in patients treated with radical cystectomy [51, 52]. However, at this time, the use of p53 as a prognostic biomarker in MIBC is still not clinically established despite over 100 studies evaluating its utility. Actually, a phase III trial designed to evaluate the benefit of stratifying organ-confined invasive BCa patients based on their p53 status for adjuvant cisplatin-based chemotherapy could not confirm the prognostic value of the p53 alteration [53].

Inactivating mutation of Rb, in conjunction with other cell cycle regulatory proteins, has been shown to be prognostic in MIBC [54]. Combined immunohistochemical assessment of p53, p21, Rb, cyclin E1 and p27 has been shown to yield predictive accuracies superior to that of any single biomarker in patients with BCa treated with radical cystectomy, and improving risk stratification by a significant prognostic margin [55–57].

As in NMIBC, apoptosis biomarkers such as survivin and Bcl-2 family are associated with outcomes in MIBC [7]. Interestingly, the proportion of specimens with survivin overexpression increases progressively from NMIBC to MIBC and to metastatic lymph node tissue [41]. In a large multicenter international validation study, addition of survivin significantly improved the accuracy of standard clinicopathologic features for prediction of disease recurrence and CSS in a subgroup of patients with pT1-3N0M0 disease [58]. Proliferation and angiogenesis biomarkers such as Ki67, VEGF and microvessel density have been associated with higher pathologic stage, lymphovascular invasion, lymph node metastasis, disease recurrence and CSS [59–62].

Finally, tissue-based gene- and transcriptome-level profiling has been used to identify biomarkers that characterize and can potentially predict prognosis in patients with MIBC [63]. Comprehensive characterization of the genomic landscape of BCa through efforts of The Cancer Genome Atlas Research Network resulted in identification of four expression clusters of high-grade MIBC [64]. Tumors in cluster I had papillary-like morphology with increased *FGFR3* expression, mutations and copy number gain, thereby suggesting that these patients may respond to FGFR inhibitors or its downstream targets. These tumors also showed decreased miR-99a and miR-100 expressions, which in turn downregulate *FGFR3* expression [65]. Tumors in clusters I and II have similar features to those of luminal A breast cancer, with high expression of luminal breast differentiation markers, including *GATA3* and *FOXA1*. These tumors also harbor increased expression of uroplakins, E-cadherin and members of the miR-200 family. Increased expression of *ERBB2* and estrogen receptor-β by these tumors also suggested that they may serve as potential targets for hormonal therapies. Expression signature of tumors in cluster III (‘basal/squamous-like’) were similar to that of basal-like breast cancers and squamous cell cancers of the head and neck and lung, characterized by overexpression of epithelial lineage genes. These findings suggest the presence of distinct molecular subtypes of MIBC with characteristic expression signatures, which may impact prognosis and serve as candidates for selective therapeutic strategies [66].

#### Blood-based biomarkers

Assessment of biomarkers in blood offers several advantages over tissue samples, such as ease of procurement, minimally invasive collection method, and higher sample homogeneity. However, clinical applicability and value of these biomarkers in the setting of MIBC remain to be further tested and validated in large well-controlled multi-institutional studies.

Biomarkers associated with cellular proliferation such as insulin growth factor binding proteins (IGFBPs) play a central role in regulating cellular growth, proliferation and transformation. IGFBP-3 is a member of this family that also has its own pro-apoptotic effects. Lower preoperative plasma levels of IGFBP-3 are a significant predictor of lymph node involvement in patients undergoing radical cystectomy and also portended a significantly increased risk of disease recurrence and cancer-specific mortality [67]. Similarly, elevated preoperative plasma levels of transforming growth factor–β1 (TGF-β1) protein have been associated with lymphovascular invasion, nodal and distant metastasis, disease recurrence and CSS [68, 69].

Inflammation and immune biomarkers such as interleukin-6 and C-reactive protein (the most widely studied serum marker for inflammation in BCa) have been consistently associated with adverse survival outcomes [70–72]. In line with observations, elevated serum levels of VEGF have also been associated with high stage and grade, lymphovascular invasion, metastases, and poor CSS [73].

The presence of circulating tumor cells (CTCs) in peripheral blood may represent an early step in the metastatic progression of BCa. In an evaluation of 55 patients undergoing radical cystectomy, the presence of CTCs was associated with survival. The study noted that patients with one or more CTCs had a shorter time to disease recurrence and shorter CSS than those with none [74]. These findings were are also found in a larger cohort of 100 consecutive patients with non-metastatic BCa undergoing radical cystectomy [75].However, despite these encouraging results, the use of blood- and tissue-based biomarkers for prediction and guidance of clinical decision-making in MIBC is still too immature due to the weak strength of evidence of published studies. Larger prospective cohorts will be important to validate these results.

## Response to systemic therapy

### Response to systemic chemotherapy

In BCa, chemotherapy can be administered intravesically (usually for NMIBC) or systemically (for MIBC and metastatic BCa). Prediction of response to chemotherapies could be helpful for decision regarding type and timing of systemic and local therapies.

A hallmark of cancer, including BCa, is dysregulation of the cell cycle which results in the sustained signal for proliferation required for cancer development. Several cell cycle regulators and markers of proliferation have been tested as predictors of chemotherapy response such as CyclinD1, CCDN1 and Ki-67. A second hallmark of cancers is their ability to escape apoptosis, a process controlled by caspases. Caspases, in turn, are regulated by several molecules involved in the detection of DNA or mitochondrial damage, including p53 and Bcl-2. p53 has been extensively tested in BCa, both as a prognostic biomarker and as a predictor of treatment response. While initial retrospective studies were promising, randomized trial results showed no role for p53 as a predictor of chemotherapy response [53, 76].

Many chemotherapy agents work by causing DNA damage and, if its DNA integrity is sufficiently disrupted, the cancer cell cannot replicate. Cancer cells that have deficient DNA damage repair mechanisms are unable to fix the damage induced by these chemotherapy agents and are, therefore, more susceptible to being killed by the agents. Proteins involved in DNA damage detection and repair that play a role in BCa chemotherapy response include BRCA-1, BRCA-2, RAD51, PARP1, ERCC1, ERCC2, ATM, RB1, and FANCC. In the study by Plimack et al., response to neoadjuvant chemotherapy (NAC) before radical cystectomy (stage T1 or less at surgery) was associated with an alteration in ATM, RB1 or FANCC [77]. Similarly, in a validation study on the role of ERCC2, ERCC2 mutation was present in 80% of patients who had pathologic stage T1 or less at time of radical cystectomy after NAC (i.e., responders) [78, 79].

It has been known for decades that the mitogenic signals derived from the binding of growth factors to their receptors are crucial for cancer development. Many growth factors and growth factor receptors signal into the cell via transmembrane tyrosine kinases, and these kinases are the targets of several new systemic therapies in oncology (such as lapatinib, pazopanib, and sunitinib). While these three drugs appear to have limited activity in BCa, the possibility of biomarker enrichment for response has been assessed [80]. Finally, microRNAs’ expression, germline single nucleotide polymorphisms, DNA ploidy, S-phase fraction (a proliferative index) and immune markers such as lymphocyte count and interleukin-8 have been preliminarily tested as predictors of chemotherapy response.

Based on the current data, none of the biomarkers has reached the level of evidence sufficient to determine therapeutic approach and resistance to targeted chemotherapies. Ongoing, biomarker-driven trials will shine more light on this important issue.

### Response to systemic immunotherapy

Over the last years, immunotherapies have proven unprecedented activity in BCa after failure of cisplatin-based therapies or in patients not suitable to receive cisplatin-based chemotherapy. Inhibition of immune checkpoints has been shown for several agents targeting programmed death-1 (PD-1) receptor or its ligand (PD-L1) and cytotoxic T-lymphocyte antigen 4 (CTLA-4). Nevertheless, the majority of patients still do not respond to treatment [81–88], which results in a significant financial burden and potential treatment-related side effects to patients who do not benefit from therapy. Therefore, biomarkers are needed to predict those most likely to benefit from checkpoint targeting therapy.

The expression of T-cell coregulatory proteins is altered in a large proportion of BCa with differential upregulation in cancer versus normal urothelium, and an association of B7-H1 with mortality after radical cystectomy in organ-confined disease has been shown [89]. Detection of PD-L1 on tumor samples with immunohistochemistry (IHC) has been used by several clinical trials to evaluate the feasibility of PD-L1 expression as a predictive biomarker. Since testing for PD-L1 is not standardized, the evaluation of PD-L1 has several limitations. The cisplatin-pretreated arm (cohort 2) of the Imvigor 210 trial revealed an association between objective response rate (ORR) to atezolizumab and PD-L1 expression status (ORR was 27% in patients with PD-L1-positive immune cells  ≥ 5% vs 18% in those with PD-L1-positive immune cells  ≥ 1% vs 15% in all patients [81]. However, in the cisplatin-ineligible arm, the ORR was independent of PD-L1 status [84]. Conversely, in the CheckMate 032 study, there was no difference in ORR between patients with PD-L1 expression  < 1% and those with PD-L1 expression  ≥ 1% (26.2% vs 24.0%, respectively) [87], CheckMate 275, evaluating nivolumab in metastatic urothelial carcinoma after platinum therapy, confirmed the association (ORR of 28.4, 23.8 and 16.1% in patients with PD-L1 expression of 5% or greater, 1% or greater and less than 1%, respectively) [82]. Finally, in KEYNOTE-045 trial, the benefit of pembrolizumab appeared to be independent of PD-L1 expression on tumor and infiltrating immune cells [83]. Spatial, time and heterogeneity are limiting factors in PD-1 biomarkers [90].

Molecular subtypes of MIBC have recently been categorized based on gene expression. The Cancer Genome Atlas (TCGA) [64] described four subtypes of BCa based on cluster analysis of messenger RNA (mRNA). The exploratory analyses from cisplatin pretreated arm of the Imvigor 210 trial showed TCGA subtypes to be independently predictive for response to atezolizumab treatment [81]. Response to atezolizumab occurred in all TCGA subtypes but was significantly higher in the luminal cluster II subtype than in other subtypes [81]. For cisplatin-ineligible patients, responses were seen across all subtypes and were more frequent with the luminal II subtype [84]. Conversely, in CheckMate 275, basal 1 subtype contained the highest proportion of responders [82].

High mutational load may be associated with better response to immunotherapy, particularly for checkpoint inhibitors, with some trials (such as Imvigor 210) showing a correlation between patients with a higher mutational burden and better responses to immunotherapeutic agents [81]. Finally, gene expression profiling (such as Interferon y gene signature) and changes in tumor microenvironment (such as chemokines and CD8 + T-cell infiltration) are promising predictors of response to immunotherapy [81, 82].

Despite these promising findings, use of biomarkers to predict the response to systemic immunotherapy remains limited; indeed, a significant proportion of patients with negative biomarkers status still respond to treatment and many patients with positive biomarkers status fail to respond.

## Conclusions

Despite the plethora of studies investigating the role of urinary, blood and tissue biomarkers in BCa with an ever-rising rate of data, none of the studies have reached the required level of evidence to change clinical practice and, therefore, none is widely used or to be recommended. Except for some specific clinical scenarios where biomarkers can be used as an adjunct in the clinical setting, use of biomarkers in BCa remains experimental and is still not recommended in clinical practice. However, biomarkers are the basis for the individualized medicine and represent undoubtedly the future of BCa treatment.
